# A simple reference state makes a significant improvement in near-native selections from structurally refined docking decoys

**DOI:** 10.1002/prot.21498

**Published:** 2007-07-10

**Authors:** Shide Liang, Song Liu, Chi Zhang, Yaoqi Zhou

**Affiliations:** 1 Howard Hughes Medical Institute Center for Single Molecule Biophysics, Department of Physiology and Biophysics, State University of New York at BuffaloBuffalo, NY 14214; 2 Department of Marine Biology, Ocean University of ChinaQingdao, 266003; 3 Indiana University School of Informatics, Indiana University-Purdue University at Indianapolis and Center for Computational Biology and Bioinformatics, Indiana University School of MedicineIndianapolis, Indiana 46202

**Keywords:** knowledge-based potential, energy score functions, reference state, binding affinity, docking decoys

## Abstract

Near-native selections from docking decoys have proved challenging especially when unbound proteins are used in the molecular docking. One reason is that significant atomic clashes in docking decoys lead to poor predictions of binding affinities of near native decoys. Atomic clashes can be removed by structural refinement through energy minimization. Such an energy minimization, however, will lead to an unrealistic bias toward docked structures with large interfaces. Here, we extend an empirical energy function developed for protein design to protein–protein docking selection by introducing a simple reference state that removes the unrealistic dependence of binding affinity of docking decoys on the buried solvent accessible surface area of interface. The energy function called EMPIRE (EMpirical Protein-InteRaction Energy), when coupled with a refinement strategy, is found to provide a significantly improved success rate in near native selections when applied to RosettaDock and refined ZDOCK docking decoys. Our work underlines the importance of removing nonspecific interactions from specific ones in near native selections from docking decoys.

## INTRODUCTION

Docking prediction refers to the prediction of the structure of a protein– protein complex from the structures of individual subunits. This is a challenging task because an unbound subunit often changes its conformation upon binding with its partner (induced fit). Docking prediction involves decoy generation and the selection of the near-native structure from decoys using a filter and/or energy function. Thus, the success of docking prediction requires an efficient method that samples complex conformations and an accurate energy function that ranks the near-native conformations as low energy conformations. Advances in sampling methods and energy functions for docking have been highlighted in several recent reviews.^1^–^13^

Various energy functions have been used in docking prediction to separate near-native structures from other structures. They are classified into two groups: “integrated” and “edge” functions based on whether or not they were used directly in sampling procedures or applied at the end of sampling procedures.^4^ Energy functions are also classified based on the methods used to obtain them. Physical-based energy functions,^14^–^17^ derived based on the laws of physics, have been applied to docking [e.g., DARWIN,^18^ DOT,^19^ Hex,^20^ Guided Docking,^21^ TSCF,^22^ SmoothDock^23^]. Some docking algorithms use semi-empirical energy functions that combine various physical terms such as surface complementarity, van der Waals interaction, generalized Born-surface area (GB/SA), and hydrogen bonding with optimized weight factors. Examples are Dock,^24^–^26^ ICM-DISCO,^27^ PPD,^28^,^29^ GRAMM,^30^ FTDOCK,^31^ 3D-DOCK,^32^ AutoDock,^33^ Surfdock,^34^ GAPDOCK,^35^ MolFit,^36^,^37^ BIGGER,^38^ Northwestern DOCK,^39^ ZDOCK,^40^ and RosettaDock.^41^ Still others use statistical energy functions derived from known protein structures.^42^–^49^ The use of energy functions is often accompanied with clustering to incorporate entropic contribution as demonstrated in recent CAPRI (Critical Assessment of PRedicted Interaction) (for example, Refs.^50^–^54^). Moreover, predicted protein–protein conformations often contain steric clashes and wrong sidechain conformations. Thus, sidechain optimization and energy minimization^31^,^55^,^56^–^59^ are important strategies for improving near-native selections from docking decoys. However, unrealistic nonspecific binding affinity proportional to the interface size is often observed for the structurally refined complexes. This makes it difficult to identify the near-native complexes with a small interface from non-native decoys with large interfaces.^60^

In this article, we extend an empirical energy function originally developed for protein design to protein–protein docking prediction. We find that this energy function together with a simple reference state provides a significant improvement in docking-structure prediction for structurally refined docking decoys. The reference state works by removing the unrealistic dependence of binding affinity of docking decoys on the buried solvent accessible surface area of interface.

## METHODS

### RosettaDock set: A refined set of docking decoys

The RosettaDock set consists of 54 protein–protein decoy sets [version 1.0 of Chen-Mintseris-Janin-Weng's benchmark^61^] downloaded from the website http://graylab. jhu.edu/docking/decoys/. The decoy sets are generated by random starting position of unbound monomer components superimposed on the native bound complex structure, followed by RosettaDock protocol to create a diffuse space distribution that covers a reasonable area (20 Å radius rmsd) with moderate density around the native position. Each decoy set has 1000 decoys/protein complex (For more detailed description, see Ref.^41^). All structures in the RossettaDock set have optimized sidechain conformations and their energies were minimized to reduce steric clashes.

### ZDOCK set: An unrefined set of docking decoys

The ZDOCK 2.3 decoy set is made of 48 protein–protein complexes (downloaded from http://zlab.bu.edu/∼rong/dock/software.shtml). Each protein complex contains 2000 docking decoys. The decoy sets were generated using fast Fourier transform (FFT) algorithm based on the PDE scoring function that combines pairwise shape complementarity (PSC) with desolvation energy (DE) and electrostatic energy (ELEC).^40^ These decoy sets are from direct docking of unbound structures without either sidechain optimization or energy minimization. The ZDOCK set is structurally refined in a procedure described below.

### Structural refinement of docking decoys from ZDOCK

#### Sidechain optimization

We used an empirical sidechain score for sidechain optimization that was originally developed for protein design.^62^ The score calculates the energy of a sidechain rotamer (*R*) of a residue, the representative conformation of the amino acid, placed on its backbone position. The score function is a linear combination of multiple energetic terms:

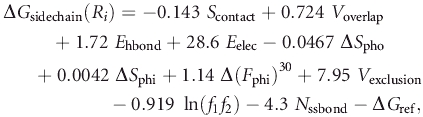
(1)
where *S*_contact_, *V*_overlap_, *E*_hbond_, *E*_elec_, Δ*S*_pho_, and Δ*S*_phi_ are atom-contact surface area, overlap volume, hydrogen bonding energy, electrostatic interaction energy, buried hydrophobic solvent accessible surface, and buried hydrophilic solvent accessible surface between the rotamer of residue *i* and the rest of the protein, respectively; *F*_phi_ is the fraction of the buried surface of non-hydrogen-bonded hydrophilic atoms; Δ(*F*_phi_)^30^ is the difference between the rotamer positioned in the protein environment and the isolated form; *V*_exclusion_ is the normalized solvent exclusion volume around charged atoms^63^; *f*_1_ is the observed frequency of the rotamer and *f*_2_ is the observed frequency of the amino acid residues in a given backbone conformation; *N*_ssbond_ is the flag of disulfide bridge (1 or 0); Δ*G*_ref_ is the reference value for the free energy difference between the rotamer in solvent and in a denatured protein.^64^ The weights of these energy terms together with the reference values (Δ*G*_ref_) were optimized so that the native residues are predicted energetically favorable over other residue types at each position of the training proteins.^62^ Here, the contact surface area (*S*_contact_) is applied to evaluate geometric complementarity between a rotamer and its protein environment^65^ while the buried solvent accessible surface area accounts for the effect of solvation. This sidechain score is used because its simplified version yields one of the most accurate sidechain prediction method.^65^

For sidechain modeling, we are only interested in the energy difference between different sidechain rotamers. Thus, the terms *f*_2_ and Δ*G*_ref_ will make no contribution for sidechain modeling because they are unchanged. They are not considered in the actual calculation. We use the backbone-dependent rotamer library developed by Dunbrack and Cohen.^66^ The updated library was downloaded from http://dunbrack.fccc.edu/bbdep/index.php. Polar hydrogen atoms, absent in the Dunbrack library, are added to calculate electrostatic interactions. The program REDUCE^67^ is used to add hydrogen atoms to all proteins.

#### Refinement algorithm

A two-step refinement algorithm is developed for docking decoys.

##### Sidechain modeling

For a given complex structure, only sidechain conformations of interface residues are optimized. Interface residues are surface residues of monomers whose solvent accessible surface areas are decreased by more than 0.1 Å^2^ upon complexation. Solvent-accessible surface area is calculated as described by Zou *et al*.^68^ Sidechains of other residues and the backbones are fixed. Sidechain conformations of interface residues are optimized by Monte Carlo simulated annealing simulation as described in Refs.^65^,^62^. First, the rotamers for the interface residues are initialized by random selections. Second, an interface residue is randomly selected and the frequency to select the residue is proportional to the number of rotamers possessed by the residue. Third, a rotamer for the residue is selected at random and the interaction energy between the rotamer and the rest of the protein 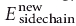 is calculated using Eq. (1). The change of rotamer for the residue is accepted if the energy value is decreased (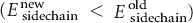). Otherwise, the change or the move is accepted with probability exp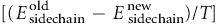. Each cycle has 50*N* trials of rotamer substitutions or 5*N* successful substitutions, whichever comes first (*N* is the number of interface residues). The annealing temperature is set to 10 initially and reduced by a factor of 0.8 after each cycle. A total of 15 cycles of annealing are conducted.

##### Energy minimization

The resulting structure from sidechain modeling is further minimized by CHARMM.^14^ This is to remove backbone steric clashes and discrete errors of the rotamers. Fifty steps of adopted-basis Newton Raphson minimizer (ABNR) are applied. All charged residues are in their charged states and distance-dependent dielectric constant is used to calculate electrostatic energy. CHARMM 19 parameters are used throughout minimization. Here, the solvent effect is only approximated by a distance-dependent dielectric constant.

In the ZDOCK decoys, only heavy atoms have coordinates. All polar hydrogen atoms are added to decoy sets with the program REDUCE.^67^ Nonpolar hydrogen atoms are ignored. To reduce the possible effect of overoptimization and computational time, only 3/4 of sidechain-optimized decoys with high binding affinity are selected for energy minimization. We discard 1/4 of decoys with the lowest predicted binding affinities after sidechain modeling. This is because those decoys usually contain severe atomic clashes and could be overoptimized if they are used in energy minimization.

### EMPIRE score function

We develop the EMPIRE score function by extending the sidechain energy described earlier for the evaluation of binding affinity. Equation (1) becomes

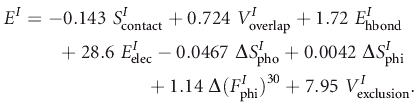
(2)

Here, each term is evaluated between two binding proteins rather than between a given sidechain and a protein in sidechain optimization. For example, 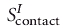 is the contacting surface area between two proteins and 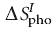 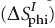 is the difference between buried hydrophobic (hydrophilic) solvent accessible surface areas before and after the binding. Both sidechain and mainchain atoms are included in calculation. Unlike Eq. (1) for protein design, the terms *f*_1_,*f*_2_,*N*_ssbond_, and Δ*G*_ref_ make no contribution to binding affinity and are not considered. However, we need to add a reference value of the protein–protein interaction to Eq. (2) instead of amino acid reference value in Eq. (1) to calculate binding free energy. Similar to the role of amino acid reference value (Δ*G*_ref_) in Eq. (1), the interface reference value is the nonspecific interaction energy between the two proteins in a refined decoy complex. Because the amino-acid reference values strongly correlate with the size of amino acid residues,^62^ we hypothesize that the interface reference value is proportional to the buried solvent accessible surface area of the interface. That is,

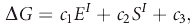
(3)
where *c*_1_, *c*_2_, and *c*_3_ are to-be-determined constants (see Results) and *S^I^* is the buried solvent accessible surface area of interface obtained with a solvent probe of radius 1.4 Å.

## RESULTS

### EMPIRE score function

We obtain the three constants (*c*_1_, *c*_2_, and *c*_3_) of the reference state [Eq. (3), methods] by multiple linear-regression analysis between *E^I^*, *S^I^*, and Δ*G*_*exp*_. The training set (1ppf, 3sgb, 1jhl, 2ptc, 1cho, 1acb, 2kai, 1vfb, 1mlc, 1nmb, 1brs, and 1ycs) are selected from 75 complexes collected by Conte *et al*.^69^ Here, a complex is selected for training if (1) its structure has a resolution of 2.5 Å or higher, (2) it does not undergo disorder–order transitions upon association, (3) its binding affinity^70^ is known, and (4) its buried interface area is between 1200 and 1600 Å^2^. The medium size of binding interface is used here because significant conformational changes between complexed and free components are observed^69^ for large interfaces of 2000–4660 Å^2^. Thus, they are not suitable for the purpose of obtaining the reference state.

We first attempted to use Eq. (3) for linear regression with the above-described training set. However, we found that Δ*G*_*exp*_ does not have a significant correlation with *S^I^*. This leads to a nearly negligible *c*_2_. Thus, we employ *E^I^* as the dependent variable in the regression, instead. This yields the equation: *E*^*I*^ = 3.09 Δ*G*_*exp*_ − 0.0295 *S*^*I*^ + 3.77. Then, a simple inversion yields *c*_1_ = 0.324, *c*_2_ = 0.00955, and *c*_3_ = −1.22. The energy unit is kilocalories per mole. The correlation coefficient between the calculated and experimental binding free energies is 0.73. The fact that *c*_2_ > 0 indicates the unfavorable contribution to association.

### Application to the RosettaDock decoy set

The EMPIRE score function is tested in the RosettaDock unbound docking decoy set of 54 protein–protein complexes. As in Ref.^41^, the selection capability of a score function is characterized by the number of structures within the five lowest energy structures whose root mean squared deviation (rmsd) values are less than 10 Å from the native complex structure (n_rmsd_). The rmsd value is calculated over the distance deviation of the *C*_α_ atoms of the smaller docking partner in the fixed coordinate frame of the larger partner. Gray *et al.* further defined that a discrimination is successful if *n*_rmsd_ is greater than or equal to three.

Without structural refinement, we find that the success rate based on *n*_rmsd_ ≥ 3 is 37/54 (69%) for EMPIRE, compared with 34/54 (63%) for RosettaDock.^41^ The success rate of EMPIRE, after 50 steps of energy minimization, increases further to 39/54 (72%). It should be noted that without the reference state, the success rate for the direct application of Eq. (2) to RosettaDock decoy sets will be 35/54. Table I compares the performance of the EMPIRE energy function for minimized RosettaDock decoys with that of RosettaDock. There are 21 targets in which EMPIRE has more near-native structures in top 5 than RosettaDock does whereas there are only 10 targets in which RosettaDock has more. The difference illustrates the significant improvement of EMPIRE over RosettaDock.

**Table I:** The Number of Top 5 Decoys with rmsd < 10 Å given by EMPIRE and the RosettaDock scoring function

Pdb ID^a^	1CGI	1CHO	2PTC	1TGS	2SNI	2SIC	1CSE	2KAI
EMPIRE^b^	1	5	4	5	5	5	5	5
RosettaDock^c^	4	3	2	5	4	5	2	4
Pdb ID	1BRC	1ACB	1BRS	1MAH	1UGH	1DFJ	1FSS	1AVW
EMPIRE	5	3	4	5	5	5	3	5
RosettaDock	1	2	4	5	5	4	5	5
Pdb ID	1PPE	1TAB	1UDI	1STF	2TEC	4 HTC	1MLC	1WEJ
EMPIRE	5	5	5	5	5	5	2	2
RosettaDock	5	5	5	5	5	5	0	0
Pdb ID	1AHW	1DQJ	1BVK	1FBI	2JEL	1BQL	1JHL	1NQA
EMPIRE	0	1	1	5	5	2	1	5
RosettaDock	5	2	5	3	5	5	1	5
Pdb ID	1NMB	1MEL	2VIR	1EO8	1QFU	1IAI	2PCC	1WQ1
EMPIRE	5	5	3	1	4	3	4	4
RosettaDock	5	5	4	1	5	0	3	3
Pdb ID	AVZ	1MDA	1IGC	1ATN	1GLA	1SPB	2BTF	1A0Q
EMPIRE	0	4	1	5	5	5	3	4
RosettaDock	0	3	2	5	1	5	4	1
Pdb ID	1BTH	1FIN	1FQ1	1GOT	1EFU	3HHR	#(≥3)^d^	#(>)
EMPIRE	0	0	4	5	2	2	39	21^e^
RosettaDock	0	0	2	0	0	0	34	10^f^

a Enzyme/Inhibitor: the first 22 protein complexes (1CGI-4HTC); antibody-antigen: the next 16 protein complexes (1MLC-1IAI); the others: (2PCC to 1A0Q); and the difficult set (1BTH to 3HHR).

b This work.

c The high-resolution RosettaDock scoring function.^41^,^71^

d The number of protein-protein complexes with more than 3 near-native structures (rmsd < 10Å) in top 5 ranked decoys.

e The number of near natives given by EMPIRE that is greater than that given by RosettaDock.

f The number of near natives given by RosettaDock that is greater than that given by EMPIRE.

### Application to ZDOCK2.3 decoy set

For the ZDOCK set, docking decoys are first refined by sidechain optimization and energy minimization. The refined structures are then ranked according to their respective binding affinities calculated by Eq. (3). The performance of the proposed refinement and scoring method is measured by success rates. Success rate is defined as percentage of test cases for which at least one near-native structure has been found within a given number (*NP*) of lowest-energy structures. Success rates for *NP* = 1 (top 1) and *NP* = 10 (top 10) are reported. According to ZDOCK and RDOCK,^40^,^59^ a near-native structure is a structure with an interface rmsd of 2.5 Å or less. An interface rmsd between a docking decoy and a native complex structure is based on the C_α_ atoms of interface residues. The value of an interface rmsd is obtained directly from ZDOCK decoy set. We do not recalculate rmsd values. This is because 50 steps of ABNR minimization makes little change in rmsd values and sidechain optimization does not change rmsd values measured by backbone C_α_ atoms.

The result for the ZDOCK 2.3 decoy set is summarized in Table II. It lists the best ranks of near-native structures in ZDOCK2.3 decoy sets along with the number of near-native structures in the 2000 decoys for a given target. Three sets of best ranks are from the application of the EMPIRE function to the original decoys, decoys with sidechain optimization and decoys with further energy minimization.

**Table II:** The Ranks and rmsd Values of Refined Structures in ZDOCK2.3 Decoy Sets

		Rank(rmsd)^c^		
Complex^a^ PDB ID#	No. of hits^b^	Original^d^	Sidechain^e^	Minimization^f^
1CGI	77	107 (1.54)	48 (2.02)	1 (2.18)
1CHO	99	1 (1.26)	1 (1.01)	1 (1.57)
2PTC	48	8 (1.03)	1 (0.44)	1 (0.44)
1TGS	109	10 (2.46)	4 (1.55)	3 (1.85)
2SNI	1	425 (2.22)	617 (2.22)	92 (2.22)
2SIC	52	2 (2.06)	3 (2.06)	3 (1.04)
1CSE	29	1 (0.50)	5 (1.10)	4 (1.24)
2KAI	16	151 (2.30)	3 (1.69)	28 (1.69)
1BRC	54	21 (1.21)	1 (1.73)	1 (2.30)
1ACB	93	2 (1.44)	14 (1.44)	4 (0.93)
1BRS	21	20 (1.30)	26 (1.97)	15 (2.29)
1MAH	28	238 (1.78)	104 (0.84)	1 (0.89)
1UGH	20	1069 (1.60)	66 (1.13)	1 (1.60)
1DFJ	51	517 (2.38)	1 (1.70)	1 (1.70)
1FSS	15	54 (1.04)	1 (1.07)	2 (1.05)
1AVW	52	1 (1.89)	12 (1.48)	1 (1.53)
1PPE^g^	393	1 (0.52)	1 (1.46)	1 (0.87)
1TAB^g^	50	1 (0.51)	1 (1.56)	1 (1.56)
1UDI^g^	35	12 (1.06)	1 (0.94)	1 (0.79)
1STF^g^	83	1 (0.80)	1 (1.42)	1 (1.01)
2TEC^g^	185	1 (0.68)	1 (1.25)	1 (0.92)
4HTC^g^	57	45 (1.40)	1 (0.69)	1 (0.69)
1MLC	17	46 (2.46)	395 (2.46)	338 (2.46)
1WEJ	22	5 (0.91)	12 (0.57)	62 (0.57)
1AHW	67	25 (1.41)	7 (1.75)	4 (1.23)
1DQJ	0	− (−)	− (−)	− (−)
1BVK	2	672 (2.34)	450 (2.34)	419 (2.34)
1FBI^g^	5	1593 (2.18)	534 (2.18)	447 (2.18)
2JEL^g^	35	598 (1.90)	20 (1.16)	1 (1.09)
1BQL^g^	70	14 (0.68)	11 (0.84)	9 (0.84)
1JHL^g^	12	121 (1.16)	9 (1.16)	50 (1.85)
1NCA^g^	67	8 (1.51)	56 (0.83)	2 (1.93)
1NMB^g^	9	1 (0.99)	427 (0.99)	337 (1.13)
1MEL^g^	71	2 (1.36)	3 (1.01)	1 (1.07)
2VIR^g^	3	79 (1.03)	527 (1.03)	521 (1.19)
1EO8^g^	2	55 (0.94)	607 (0.94)	72 (0.94)
1QFU^g^	18	21 (0.75)	92 (0.78)	1 (0.78)
1IAI^g^	3	52 (1.47)	106 (1.47)	429 (1.70)
2PCC	0	− (−)	− (−)	− (−)
1WQ1	54	121 (2.23)	10 (1.88)	9 (1.20)
1AVZ	0	− (−)	− (−)	− (−)
1MDA	0	− (−)	− (−)	− (−)
1IGC^g^	3	141 (1.18)	785 (1.20)	227 (1.18)
1ATN^g^	24	1 (0.56)	1 (0.52)	1 (0.80)
1GLA^g^	0	− (−)	− (−)	− (−)
1SPB^g^	112	2 (0.61)	1 (0.61)	1 (0.95)
2BTF^g^	35	1 (0.65)	1 (1.02)	1 (0.83)
1A0O^g^	4	21 (2.45)	13 (2.25)	427 (2.45)
Top 1 (Top 10)		10 (18)	14 (22)	20 (29)

a Enzyme/Inhibitor: the first 22 protein complexes (1CGI-4HTC), antibody-antigen: the next 16 protein complexes (1MLC-1IAI). The rest are 10 other complexes.

b The number of hits (near-native structures with interface rmsd < 2.5Å).

c The highest rank of hits (and its interface rmsd).

d Original decoys without any refinement.

e Results after sidechain optimization.

f Results after sidechain optimization and energy minimization.

g Docking decoys from unbound and bound structures.

Near-native structures of 10 proteins (out of 43 proteins with near-native structures, 23%) are ranked as top 1 in the direct application of the scoring function to the ZDOCK 2.3 decoy set. The number of correctly ranked near-native proteins increases to 14 (33%) after sidechain optimization and 20 (47%) after further energy minimization. The number of near-native structures that are ranked within top 10 also increases from 18 (42%) for the original, 22 (51%) for sidechain optimizied, to 29 (67%) for energy-minimized decoys. This highlights the importance of sidechain optimization and energy minimization.

The success rates for enzyme/inhibitor group are more impressive. In a total of 22 targets, there are 11 (50%) and 14 (64%) complexes whose near-native structures are ranked number 1 after sidechain optimization and after energy minimization, respectively. For the 16 antigen-antibody complexes, however, none of their near-native structures are successfully ranked as number 1 after sidechain modeling and only three after energy minimization. Among the 10 other targets, 4 do not contain any near-native structures. The success rates are 50% before and after energy minimization for the remaining six targets.

The main reason behind different success rates for different types of complexes is that the enzyme/inhibitor group has significantly more near-native structures per target in 2000 decoys than those of antigen-antibody complexes. In fact, for all targets with 24 or more near-native structures in their decoys (1.2%), there is at least one near-native structure ranked within top 10 after energy minimization. This is true regardless of actual type of complex structures (enzyme/inhibitor, antigen-antibody, or others). Thus, the success rate is largely determined by the quality of docking conformations (i.e., the population of near-native structures).

**Figure 1:**
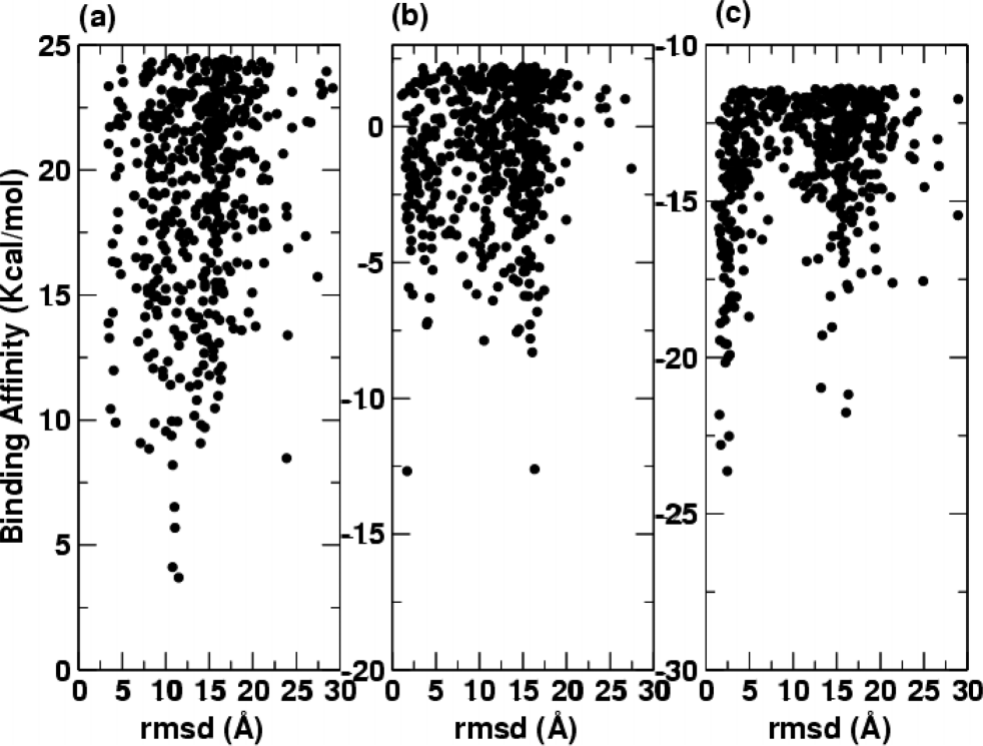
The binding affinity as a function of rmsd (Å) for the original ZDOCK decoys (a), after sidechain optimization (b), and after 50 steps of minimization(c). Only top 500 ranked decoys for each case are shown in this figure. This is the result of target 1DFJ.

To further illustrate the importance of refinement, one example for the ribonuclease A/ribonuclease inhibitor complex (1dfj) is shown in Figure 1. Before any structural refinement, strong atomic clashes make the binding free energies of all docking decoys positive [Fig. 1(a)]. Sidechain optimization removes most steric clashes and majority of docking decoys now have negative (attractive) binding free energies [Fig. 1(b)]. Most near-native structures, however, do not yet distinguish significantly from other decoys. Only further energy minimization [Fig. 1(c)] makes a clear identification of near-natives as a cluster of structures that are separated from the rest.

**Figure 2:**
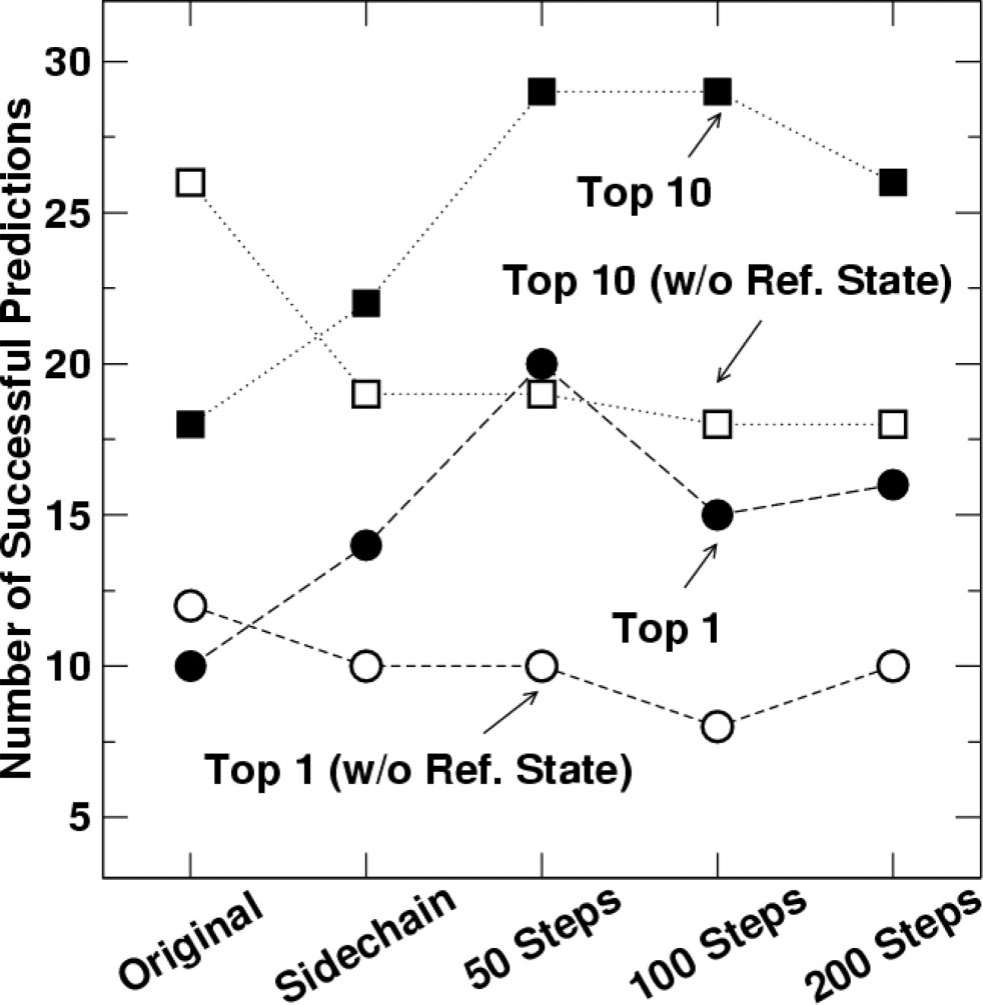
The number of successful predictions with or without the reference state as labeled for original ZDOCK decoys, after sidechain optimization, after further 50, 100, and 200 steps of minimization.

The reference state used in EMPIRE plays an essential role in the accuracy of EMPIRE. Figure 2 compares the performance made by EMPIRE with or without the reference state for the original ZDOCK decoys, decoys after sidechain optimizations, and decoys after further 50, 100, and 200 steps of energy minimization. It is clear that the use of the reference state leads to a significant increase in the number of successful predictions for the refined complexes. Another interesting result is that 50 steps of minimization lead to the highest success rate for including near-native structures within the top 10, in particular. We found that 50 steps of minimization yield the binding affinities of near-native structures close to that of experimental-binding affinity whereas 100 or 200 steps of minimization produces the value of binding affinity that is much lower than the corresponding experimental value (20 of 48 targets have known binding affinities).^41^ Sixteen of the 20 targets have native like complexes in the decoy sets and the mean value of their experimental-binding free energy is −12.6 kcal/mol. This is essentially the same as the calculated value, −12.7 kcal/mol, of the best ranked near-native decoys of the 16 targets after minimized 50 steps. The corresponding values after 100 and 200 steps are −15.8 and −20.3 kcal/mol, respectively). This explains the highest success rate made by 50 steps of minimization because the parameters of the reference state in EMPIRE are trained by experimental-binding affinities. In other words, a stronger interface reference value would be needed to counter the over-reduction of energy for 100 or 200 steps of minimization (for details, see Discussion).

It should be emphasized that the parameters in the reference state are trained by the native structures and experimental-binding affinities. A 50-step minimization is the best minimization protocol for decoy refinement because it produces binding affinities similar to experimental-binding affinities. However, one certainly can optimize parameters based on minimization protocol as well (see Discussion).

**Figure 3:**
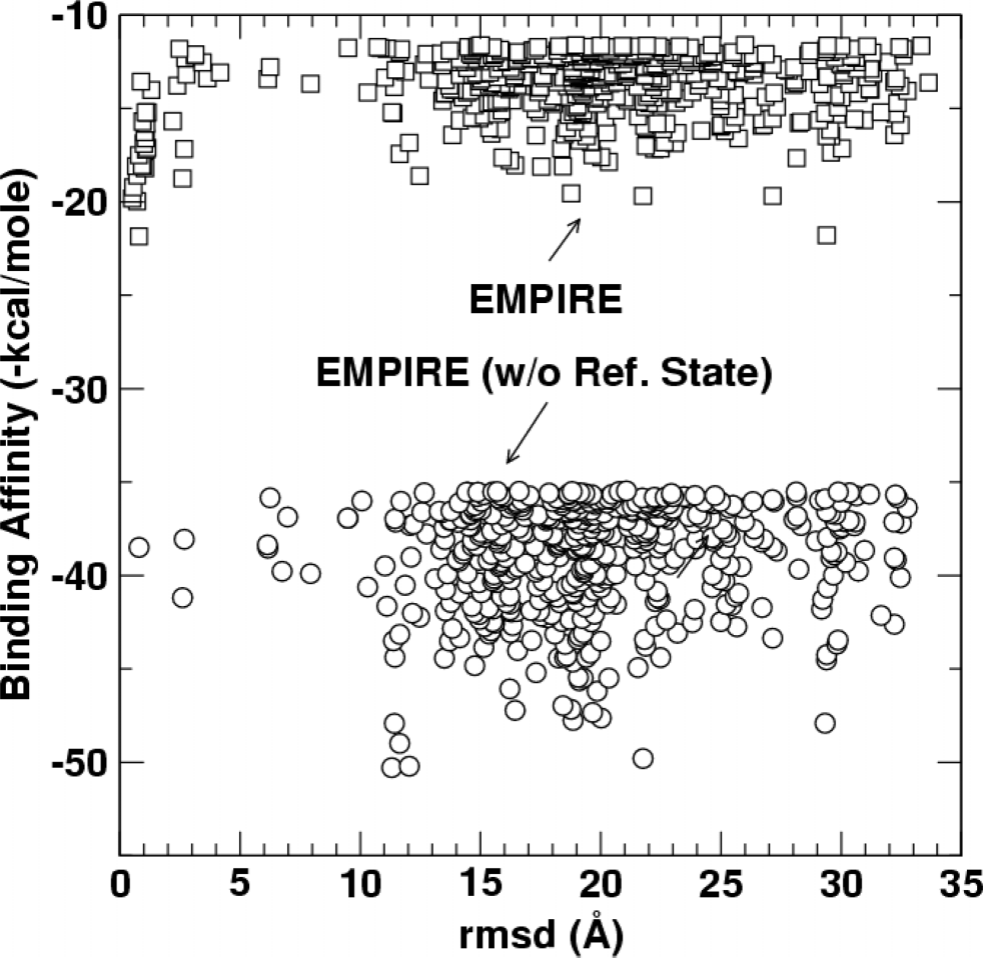
As in Figure 1, but compares the binding affinity for EMPIRE with or without the reference state (target 1ATN). Only top-ranked 500 decoys are shown.

**Figure 4:**
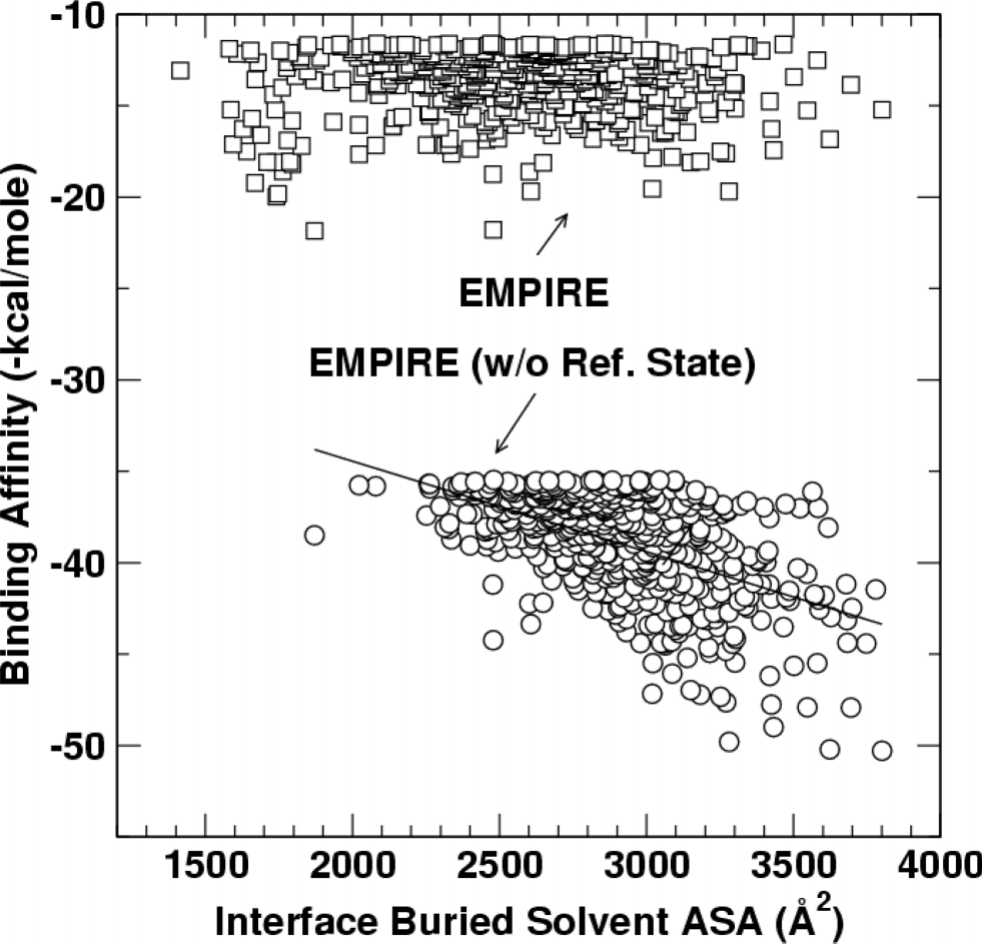
The binding affinity as a function of the buried solvent accessible surface area of interface for EMPIRE with or without the reference state (target 1ATN). Only top-ranked 500 decoys are shown. The solid line denotes the result from linear regression on the data given by EMPIRE without the reference state (with a correlation coefficient of −0.54). There is no correlation for the data given by EMPIRE (with the reference state).

To further illustrate the importance of the reference state, we plot the binding affinity as a function of rmsd (Fig. 3) and buried solvent accessible surface area of interface (Fig. 4) for target 1ATN. In the absence of reference state, near-native structures (rmsd < 2.5 Å) do not have low-binding free energies. The lowest energy conformations have rmsd values that are greater than 10 Å. After incorporating the reference state in EMPIRE, near-native structures become a part of low energy conformations. Figure 4 further shows that there is a correlation between the buried solvent accessible surface area and binding affinity for EMPIRE without reference state. The correlation coefficient is −0.54. The use of the reference state in EMPIRE effectively removes this correlation (the correlation coefficient becomes 0.02). Thus, removing the unrealistic dependence of binding affinity on the buried solvent accessible surface area of interface is the main reason for the success of EMPIRE.

## DISCUSSION

In this article, we successfully construct an empirical energy function for docking prediction by adding a simple reference state to a scoring function originally developed for protein design. The new scoring function, called EMPIRE, is tested in RosettaDock with or without further energy minimization. The success rates (3 or more in top five ranked decoys that are “near-native”) are 69% with the original decoys and 72% with further energy minimization, respectively. This can be compared to 63% made by RosettaDock.^41^,^71^

EMPIRE is further tested in ZDOCK 2.3 decoy set. It successfully ranks 20 near-native complex structures in top 1. We have also applied EMPIRE to the ZDOCK 2.1 decoy set. This leads to 14 successful predictions. The reduction of the number of successful predictions is expected because the ZDOCK 2.1 decoy set has a much smaller number of near-native-structures (27 per complex) than the ZDOCK 2.3 decoy set (46 per complex in average). On the other hand, RDOCK, a structural refinement and scoring protocol, performs much better on the ZDOCK 2.1 decoys than on the ZDOCK 2.3 decoys.^59^ The number of successful prediction for RDOCK+ZDOCK 2.1 (top 1) is 18, compared to 20 for EMPIRE+ZDOCK 2.3.

For each target, a higher number of near-native structures corresponds well with the improved ability of EMPIRE in detecting near-native structures. In fact, for all targets with 24 or more near-native structures in their decoys (1.2%), there is at least one near-native structure ranked within top 10 after energy minimization. This is true regardless if a complex is an enzyme-inhibitor, antibody-antigen, or other complex. That is, a lower success rate in ranking near-native structures of antibody-antigen complexes than that of enzyme-inhibitor complexes reflects a smaller number of near-native structures in ZDOCK docking decoy sets of the former complexes. This suggests the robustness of the EMPIRE energy function in identification of near-native structures. It is of interest to note that RosettaDock is somewhat better than EMPIRE in detecting near-native structures for antibody-antigen complexes (EMPIRE recognizes more near-native structures within top five in four antibody-antigen complexes and less so in six complexes than RosettaDock, Table I). This may be related to the fact that many of these antibody-antigen complexes are used in training the weights of energy terms of RosettaDock.^41^,^71^

The success of the EMPIRE score function highlights the importance of removing nonspecific interactions. Proteins interact with each other via recognizing specific interfaces, rather than according to the size of interfaces. However, an unrealistic correlation between binding affinity and the interface area is often observed for structurally refined complexes (Fig. 4). This unrealistic correlation is possibly caused by local energy minimization with an approximate energy function. In general, more minimization leads to higher binding affinity. Although binding affinities of near-native decoys may increase faster than those of non-native decoys with a similar interface area during minimization, binding affinities of decoys with a large interface will increase even faster. EMPIRE attempts to remove this artifact empirically by subtracting an interface reference state.

The removal of the unrealistic dependence of binding affinities on interface areas is an empirical approach. One issue with this approach is that the performance of EMPIRE will depend on refinement protocol. This is because longer energy minimization will further increase binding affinities of docking decoys and the rate of increase depends on interface areas and other factors. As a result, EMPIRE will work best with a fixed minimization step that leads to a nativelike binding affinity. This highlights the approximate nature of the EMPIRE energy function. The RDOCK refinement protocol^59^ obviously has the same problem. The number of steps in its refinement procedure is precisely defined.

We examined the dependence of reference parameters on minimization steps. This is done by varying *c*_2_ in Eq. (3) at an interval of 0.001 to make the best near-native prediction for docking decoys with different refinement procedures. The best performances are a total of 14, 21, 20, and 24 targets successfully predicted (*NP* = 1) after sidechain modeling, and sidechain modeling plus 50, 100, and 200 steps of energy minimization, respectively. Thus, optimizing *c*_2_ can increase the prediction success rate for decoys refined by 100 or 200 steps of energy minimization, compared to *c*_2_ obtained from native structures and experimental-binding affinities. As a result, a stronger interface reference value would be needed to counter the increase of binding affinities for over optimized decoy sets. Indeed, we find that the best *c*_2_ value for the decoy set refined by 200 steps of energy minimization yields binding affinities that are close to the experimental values. Although this value yields the highest success rate (24 targets), we prefer 50 steps of minimization and the parameters independently generated from native structures and experimental-binding affinities. This is to avoid overoptimization.

**Figure 5:**
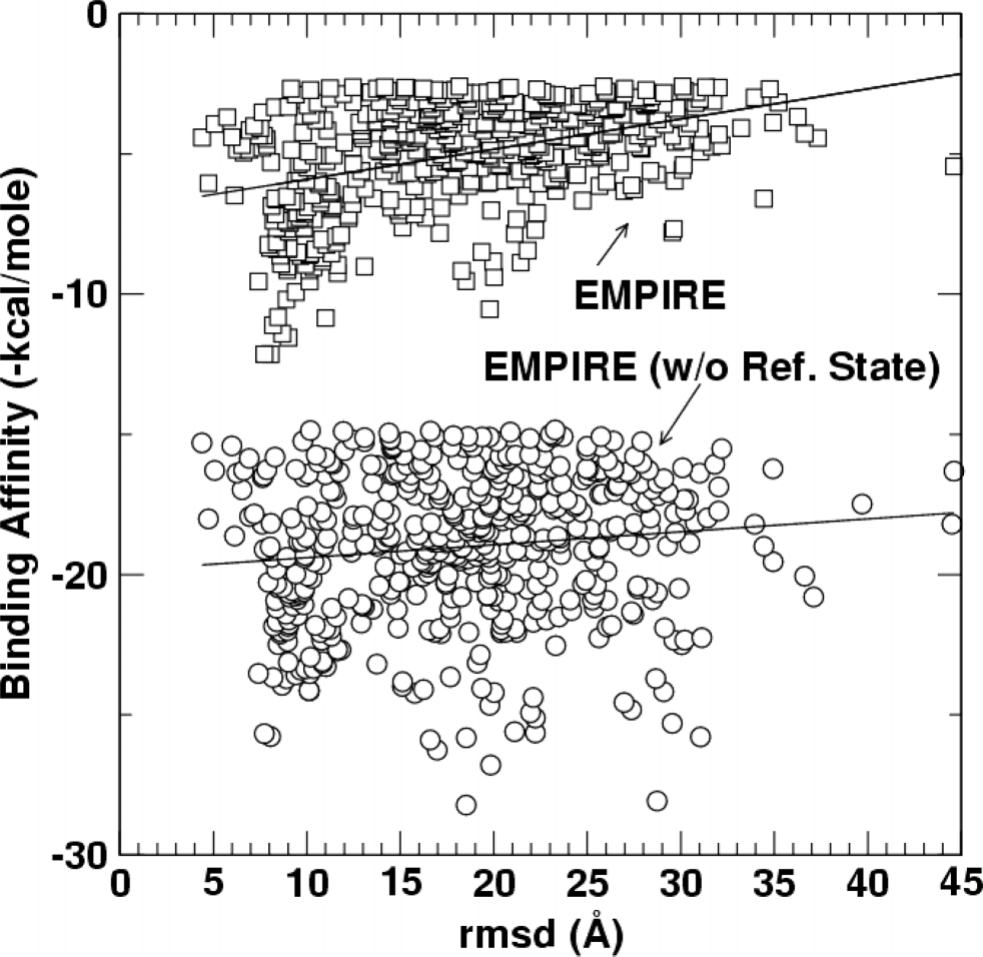
As in Figure 1, but compares the binding affinity for EMPIRE with or without the reference state (target 1GOT). Only top-ranked 500 decoys are shown. The solid lines denote the result from linear regression on the data with a correlation coefficient of 0.12 for EMPIRE without the reference state and 0.41 for EMPIRE.

One interesting result is that EMPIRE performs well for some difficult targets (1BTH to 3HHR in Table I). We found that this is mainly because of the improvement of correlation between rmsd values and energy scores after the introduction of the reference state. Figure 5 displays the rmsd values of decoys as a function of their energy scores with and without the reference state in EMPIRE for a difficult target 1GOT. The correlation coefficient between rmsd values and energy scores increases from 0.12 to 0.41 after the reference state is used in EMPIRE. Without the interface reference state, the selected decoys with the lowest interaction energy have a overwhelmingly larger interface than the near-native docking decoys.

Some docking decoys were made from the docking between an unbound structure and a bound one (Table II). EMPIRE performs better on those targets than the decoys from unbound–unbound docking. For example, near-native structures for unbound–bound-docked enzyme-inhibitor targets are all ranked number 1. The difference between unbound-unbound and unbound-bound docking is largely because there are more near-native structures in unbound-bound decoys.

The most time-consuming part of calculations in this study is sidechain optimization via simulated annealing. Sidechain modeling for 2000 decoys typically takes 1–3 weeks on a single 2.6 GHz AMD Opteron CPU. We use our in-house sidechain optimization since its simplified version is one of the most accurate sidechain prediction algorithms.^65^ For sidechain modeling, EMPIRE is 1–2 times slower than its simplified version but the prediction accuracy is similar. In this study, we use the same energy function for intraprotein and interprotein interactions (except the reference state) that allows a consistent evaluation of the energy function.

An executable version of the EMPIRE score function and its corresponding webserver are freely available at http://sparks.informatics.iupui.edu.
